# Benthic colonization in newly ice-free soft-bottom areas in an Antarctic fjord

**DOI:** 10.1371/journal.pone.0186756

**Published:** 2017-11-08

**Authors:** Cristian Lagger, Natalia Servetto, Luciana Torre, Ricardo Sahade

**Affiliations:** 1 Universidad Nacional de Córdoba, Facultad de Ciencias Exactas, Físicas y Naturales, Laboratorio de Ecología Marina, Córdoba, Argentina; 2 Consejo Nacional de Investigaciones Científicas y Técnicas (CONICET), Instituto de Diversidad y Ecología Animal (IDEA), Córdoba, Argentina; Sveriges lantbruksuniversitet, SWEDEN

## Abstract

Extended glacier retreat is among the main consequences of the rapid warming of the West Antarctic Peninsula. Particularly, in the inner part of Potter Cove (South Shetland Islands, Antarctica) large areas are now exposed to open sea conditions owing to the retreat of Fourcade glacier. During the 2010 austral summer, underwater photographic surveys were undertaken by SCUBA diving up to 30 m in these new ice-free areas 80 m from the glacier front. Our main aim was to investigate colonization and early succession of the benthic assemblages on soft-bottom areas. Here, we reported a total of 1,146 animals belonging to 13 taxa. Filter-feeders comprised the largest trophic group and sessile fauna showed much higher coverages and densities than mobile fauna at all depths. The most abundant groups were ascidians and bryozoans, which together comprised ~90% of all taxa documented. In a region where most of marine-terminating glaciers are in retreat, these results are an important contribution to improve our knowledge on colonization in the newly ice-free areas.

## Introduction

Southern Ocean benthic fauna, including those found beneath the extent of existing ice shelves or inhabit the former extent of the ice shelf, is much more diverse and complex than expected [1–5]. These marine ecosystems are currently threatened by the changing environmental conditions; especially along the Western Antarctic Peninsula (WAP), which is one of the regions where warming is proceeding more rapidly on Earth [6–9]. Massive ice losses, represented by ice-shelf collapses and sea-ice reduction are among the main impacts of this rapid regional warming [10–12]. Furthermore, estimations of ice mass change suggest that glaciers in this region are particularly sensitive [13], and an accelerated mass loss has occurred during the last 60 years [14,15]. A recent Antarctic Peninsula glacier basin inventory indicates that 90% of the studied marine-terminating glaciers have been reduced in area since the 1940s [16].

The loss of ice coverage drives hydrological modifications on coastal regimes that may negatively affect pelagic and benthic communities [17–19]. This loss of ice also opens up new areas for biological productivity and benthic colonization [20–22].Then, this in turn could drive to positive or negative feedbacks on climate change [20,23], depending on the prevalence of either one or the other process.

The slow growth rates and population turnover registered in natural and artificial substrates [24–29], together with the relative constancy of environmental variables prevailing in Antarctic ecosystems, has led to an image of a certain stability in structural patterns and a low speed in ecological processes in the Antarctic benthos [6,30,31]. However, in the last years there have been also reports of rapid responses, fast colonization and growth rates of some species [3,21,32–35]. In addition, we also documented in a rocky island, recently uncovered by glacier retreat at Potter Cove, a benthic assemblage characterized by high species richness, diversity and structural complexity [22]. These finding challenge the extended idea of slow colonization processes in Antarctica.

Antarctic benthic colonization studies in newly ice-free areas due to glacier retreat are extremely limited as well as glacier retreat effects (e.g. ice scouring, high sedimentation rates, large salinity shifts, etc.) on benthic systems. Then, the progressive retreat of Fourcade glacier in Potter Cove (up to 1 km since the 1950s, [36]) is not only a good example of the observed situation in the majority glaciers on the WAP but also provides an opportunity to examine these newly ice-free areas and the ongoing ecological processes. Considering that the estimated area affected by changes in the extension of tidewater glaciers is around 2.97 × 10^6^ km^2^ [37], information on these processes is necessary.

Our main aim was to investigate the colonization and early succession of the benthic assemblages in new ice-free soft bottom areas. We used seafloor photographic surveys to register the benthic assemblages on recently uncovered soft substrates. As far we know, is the closest photographic register to a glacier termini in Antarctica. In this context, we also discuss our results with recently reported findings at the new ice-free rocky island of Potter Cove. These results will provide important insights into the benthic ecosystems responses to the current climate change experienced along the WAP as well as the registered around Antarctica.

## Materials and methods

### Study area

**Fig 1:**
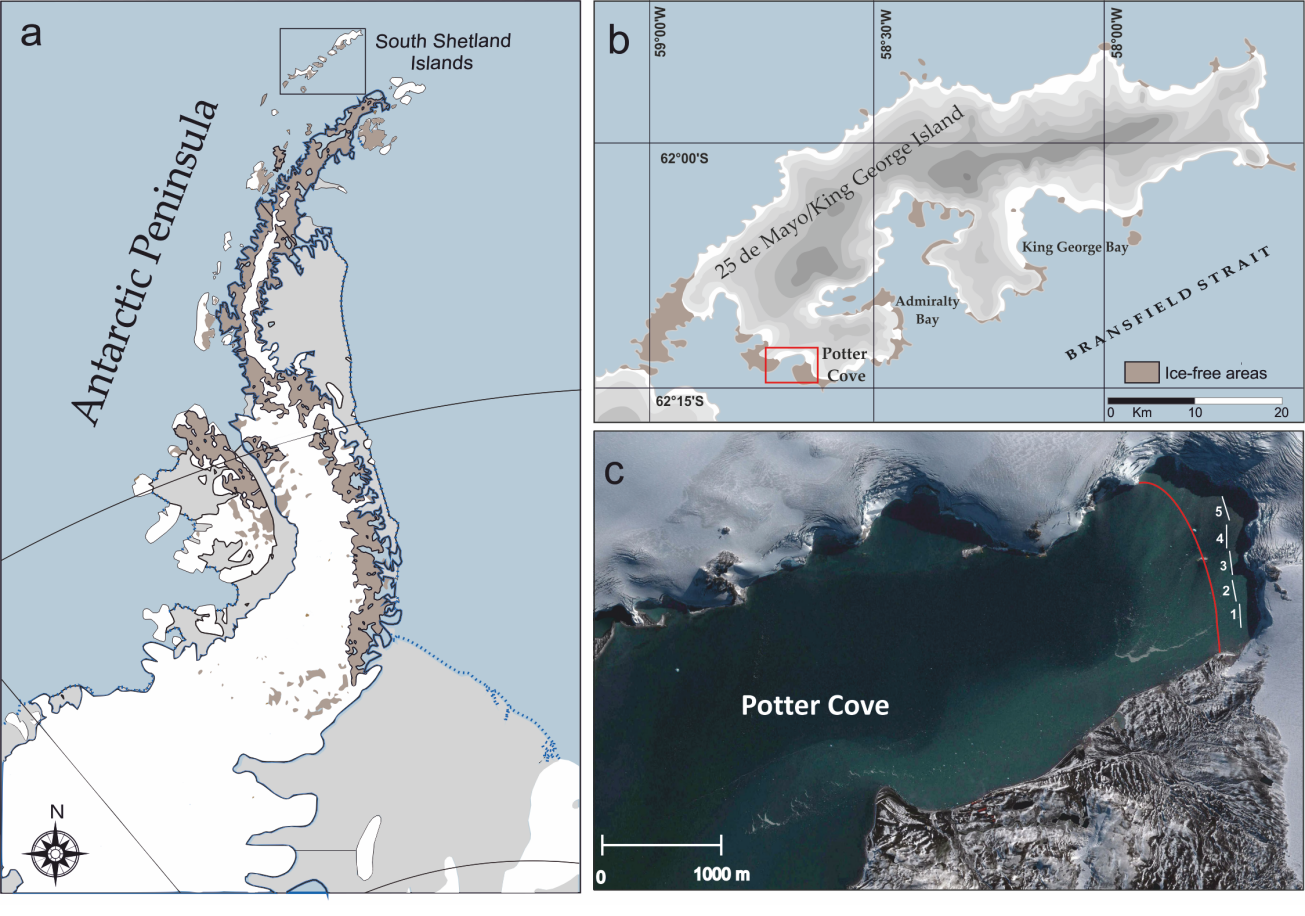
Location of the area of investigation. **(a)** Map of the location of South Shetland Islands on the Antarctic Peninsula (black square). **(b)** Location of Potter Cove in 25 de Mayo/King George Island and **(c)** satellite image of Potter Cove (Google Earth, 2013). The red line marks the approximate position of the Fourcade Glacier in 2000. At each depth profile (1 = 10 m, 2 = 15 m, 3 = 20 m, 4 = 25 m, 5 = 30 m) 45 photographs were randomly taken.

Potter Cove (62°14′ S, 58°38′ W), where the Argentine Antarctic Station Carlini (formerly Jubany) is located, is a small fjord-like cove in the South Western end of 25 de Mayo/King George Island (South Shetland Islands, WAP, Fig 1). The cove opens into Maxwell Bay which connects to Bransfield Strait. A shallow sill (<30 m) separates the inner and outer cove sections. The inner cove is characterized by soft sediments and shallower depth (<50 m) than the outer cove, where the bottom is mainly rocky and depths are >100 m. Air temperatures are typical of the maritime West Antarctica Peninsula, with moderately cold winters (−6.3°C) and warmer summers (+2.5°C). The cove experiences average sea surface temperatures around 1°C during summer months. A significant increase was observed in both mean air temperatures and sea surface temperature in the past two decades in Potter Cove [38]. Further details on the hydrographical characteristics and other environmental conditions of the cove have been recently described by Schloss et al. [38].

Glacier retreat has been proceeding continuously for the past few decades in several tributaries embayments within the Maxwell Bay, including Potter Cove [36]. The tidewater glacier that surrounds Potter Cove (Fourcade glacier) showed a remarkable retreat of several hundred meters in the last decades [36]. The glacier calving introduces large volumes of meltwater and small pieces of ice into the cove throughout the summer months [39–41]. This progressive glacier retreat opens new ice-free areas in the cove for colonization and succession, which offers excellent opportunities to study these processes under natural conditions [21,22]. With the exception of two recently uncovered rocky islands, these new ice-free areas are dominated by soft bottoms mainly composed by silt and clay. Sediment accumulation rates in these areas are also high compared with the other areas of the cove [38].

### Sampling design and data analysis

The study was conducted close to the Fourcade glacier front (<80 m), in the new ice-free areas at 10, 15, 20, 25 and 30 m depths. Photographic surveys were undertaken by SCUBA diving during the austral summer from December 2009 to February 2010. A high definition SONY SR-12 digital camera housed in an Amphibico case and fitted with 2 led lights was used to take the pictures. An aluminum frame (40 x 30 cm) was attached to the housing and used to quantify the sampled area. At each depth profile, photographs were randomly taken along the selected isobaths. A total of 45 0.12 m^2^ images were taken at each depth, resulting in a total area of ~30 m^2^. High definition videos along of the new ice-free areas were also recorded as extra supplementary material for taxonomic identification.

Photographs were projected onto grids of 100 points and those underlying each organism were counted to estimate percentage cover and bare substratum per square meter. Animals in each photograph were counted (abundance) and the total number divided by the area sampled to estimate densities. Abundance and percentage cover were analyzed from the photographs with ImageJ. Identification and quantification was always conducted by the same person (CL) in order to reduce methodological bias. All abundance values in the text are given as mean ± SE. The resolution of images was sufficiently fine to detect and identify organisms as small as ~10 mm in diameter. Burrowing infauna that could be recognized from the visible portions of their bodies (e.g., burrowing bivalves such as *Laternula elliptica*) were also included. All discernible fauna were identified to the lowest possible taxonomic level, which was generally species (although bryozoans and some sponges could not always be identified to this level). Some components of the biota were excluded from the analysis: encrusting taxa (some smaller bryozoans and terebellid polychaetes) could not be accurately identified and quantified. For some analysis, faunal components were classified into two groups: mobile and sessile animals.

Diversity across different depths was compared using the Shannon-Wiener index *H'*, Pielou’s evenness *J'* and cumulative k-dominance curves. To test for significant differences among depths, a jackknife procedure was used to obtain pseudovalues that allowed the estimation of mean and variance and the use of ANOVA [42]. The Levene test was used to check for variance homogeneity and a Student-Newman-Keuls *post hoc* procedure to detect depths that significantly differed. To assess structural patterns of the assemblages and to analyze vertical and horizontal spatial variations a series of multivariate analyses were performed. A similarity matrix using Bray-Curtis distance was constructed after a square-root transformation (in order to reduce dominance weight) of percentage cover of the species. The similarity matrix obtained was used to perform descriptive classification and ordination analyses. A cluster analysis using the unweighted pair group method average (UPGMA) to construct the dendrogram was used as a classification analysis, and a non-metric multidimensional scaling (nMDS) was used as an ordination technique. To facilitate visualization and interpretation of cluster and nMDS analyses the original 45 samples per depth were pooled (9 consecutive images) resulting in 5 replicates. Then, to test for statistical differences among *a priori* defined assemblages (defined by depths), the same similarity matrix was used to perform a one-way Analysis of Similarities (ANOSIM). The nature of community grouping identified in the MDS ordinations was further explored using the similarity percentages program (SIMPER) to determine the contribution of individual species to the dissimilarity average between samples [43]. A cut-off point of 75% of total dissimilarity between groups was used. All analyses were performed using the software PAST 2.1 [44], except the diversity indices, which were performed with Infostat [45].

In the framework of the Research project PICTO Antártida Ref 36326. ANPCyT-DNA (“*Antarctic benthic communities*: *an interdisciplinary approach to analyze the possible impact of global warming*”), the Environmental and Tourism Antarctic Management Program of the National Direction of the Antarctic (Dirección Nacional del Antártico) in the Republic of Argentina, has issued the appropriate permission to all the stages of this research:

- To the Specially Protected Area N° 132 ‘‘Peninsula Potter” (under art. 7, Annex V of the Madrid Protocol, Law 25260).

This permission properly followed the regulations in force.

## Results

Taxonomic compositions and abundances classes of the epibenthic faunal community in the new ice-free soft bottom area of Potter Cove are summarized in Table 1. A total of 1,146 animals belonging to 13 taxa and 8 phyla were identified. Sessile fauna showed much higher coverages and densities than mobile fauna at all depths.

**Table 1:** Taxonomic list and abundances classes of taxa present in the new ice-free soft bottom area at Potter Cove.

Phylum	Class	Taxon	Depth (m)				
			10	15	20	25	30
Porifera	Demospongiae	*Haliclona* sp.3	●		●		
		*Haliclona* sp.2		●		●	
Cnidaria	Anthozoa	*Hormosoma scotti *	●				
Mollusca	Bivalvia	*Neobuccinum eatoni*	●				●
		*Laternula elliptica*	●				
Ctenophora	Tentaculata	*Lyrocteis flavopallidus*	●		●		
Bryozoa	Gymnolaemata	*Cellaria* sp.			●●	●●●	●●
Arthropoda	Malacostraca	*Serolis* sp.	●	●●●			●
Echinodermata	Asteroidea	*Odontaster validus*	●	●	●	●	
Chordata	Ascidacea	*Sycozoa sigillinoides*					●
		*Corella antarctica*	●●	●●●	●●●	●●	
		*Cnemidocarpa verrucosa*	●●●	●●	●●●	●●●●	●●●
		*Molgula pedunculata*	●●●	●●	●●●	●●●●	●●

Abundances classes at each depth are represented by

● ≤10 ind.

●●11 to 50 ind.

●●●51 to 100 ind.

●●●● 101 to 360 ind.

**Fig 2:**
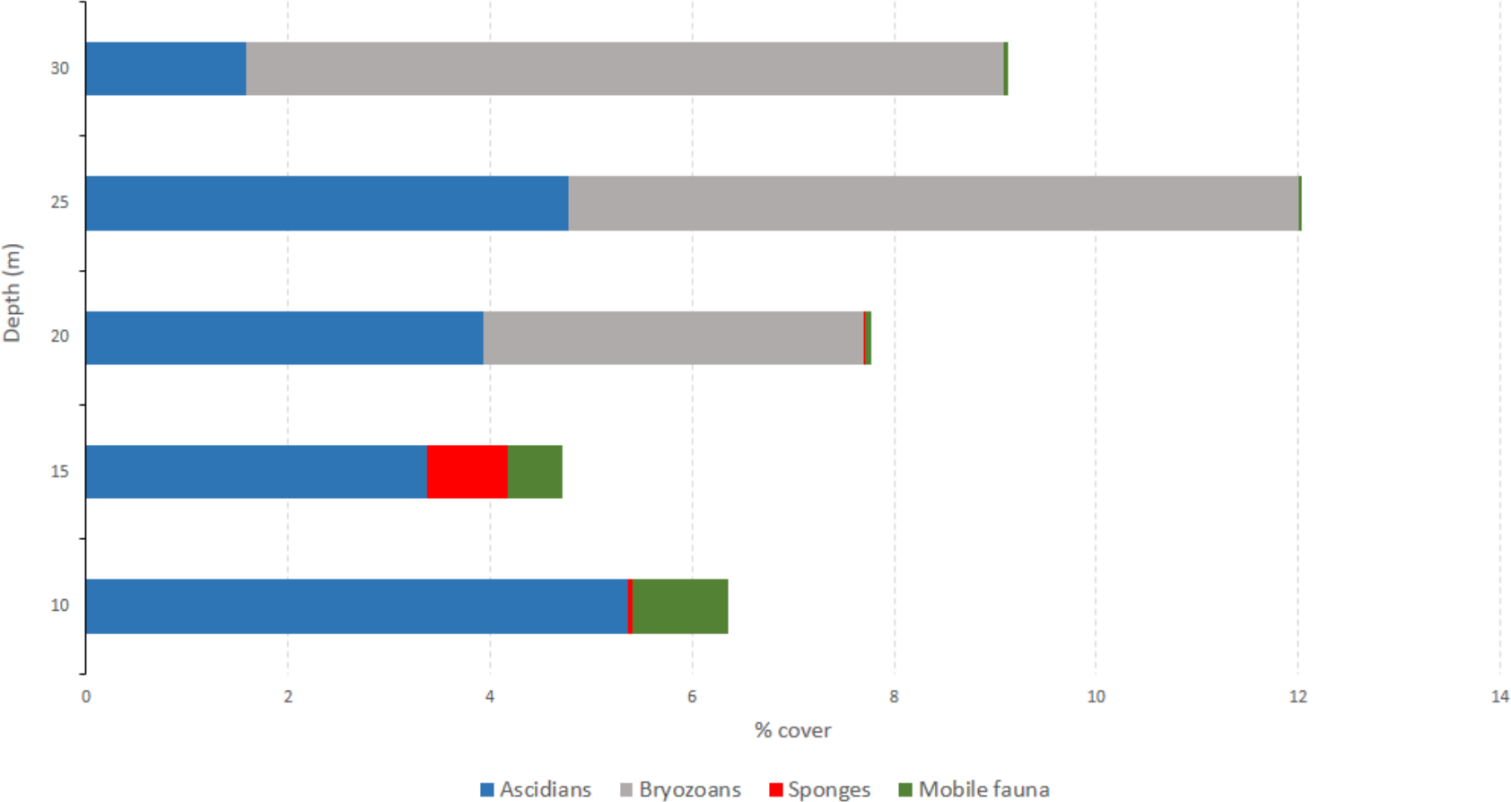
Mean percentage cover of the major taxa present at each depth in the new ice-free areas at Potter Cove. Note that all mobile species were grouped together under "Mobile".

The percentage cover of the major taxa and density of each species along a bathymetric gradient are shown in Fig 2 and Table 2 respectively. The five species of mobile fauna represented only <10% (8.78%) of the total faunal abundance. Dominated numerically by the isopod *Serolis* sp. and the seastar *Odonstaster validus*, mobile fauna showed a peak of abundance at 10−15 m, and then decreased sharply with depth reaching values close to zero at 20, 25 y 30 m. Percentage of bare substratum was very high at all depths (>89%).

**Table 2:** Density of each species along the bathymetric gradient sampled in the new ice-free areas at Potter Cove (mean ± SD, *n =* 45).

Depth (m)	C. v.	M. p.	C. a.	S. s.	*Cellaria* sp.	Porifera	L. e.	H. s.	O. v.	N. e.	*Serolis* sp.	L. f.
**10**	11.11 ± 15.49	10.37 ± 17.6	3.89 ± 8.81	−	−	0.19 ± 1.24	1.48 ± 3.22	0.19 ± 1.24	1.48 ± 3.22	0.93 ± 3.19	0.56 ± 2.1	0.56 ± 2.1
**15**	6.67 ± 13.01	4.44 ± 12	10.74 ± 19.1	−	−	0.56 ± 2.1	−	−	1.3 ±3.53	−	12.96 ± 17.99	−
**20**	10 ± 17.37	11.3 ± 22.48	13.15 ± 48.64	−	6.3 ± 9.76	0.19 ± 1.24	−	−	0.19 ± 1.24	−	−	0.19 ± 1.24
**25**	23.15 ± 37.93	34.07 ± 39.52	9.63 ± 17.13	−	12.22 ± 9.67	0.19 ± 1.24	−	−	0.19 ± 1.24	−	−	−
**30**	11.3 ± 21.26	5.19 ± 16.5	−	0.19 ± 1.24	8.15 ± 9.48	−	−	−	−	0.19 ± 1.24	0.19 ± 1.24	−

Densities (inds.m^-2^) of all species found at each sampled depth profile (C. v.: *Cnemidocarpa verrucosa*, M. p.: *Molgula pedunculata*, C. a.: *Corella antarctica*, S. s.: *Sycozoa sigillinoides*, *Cellaria* sp., Porifera, L. e: *Laternula elliptica*, H. s.: *Hormosoma scotti*, O. v.: *Odontaster validus*, N. e.: *Neobuccinum eatoni*, *Serolis* sp., L. f.: *Lyrocteis flavopallidus*).

The most speciose and abundant group were ascidians, which were represented by four species, three solitary and one colonial, *Sycozoa sigillinoides*. Ascidiacea represented 77.4% of all species, followed by bryozoans. Together, these major groups comprised ~90% of all taxa documented. *Molgula pedunculata* and *Cnemidocarpa verrucosa* were the most abundant species with a mean density of ~13 inds.m^-2^ (13.07 ± 1.72 and 12.44 ± 1.55 respectively), followed by other ascidian species, *Corella antarctica* (7.48 ± 1.67 inds.m^-2^). Bryozoans also registered one of the higher mean density with 5.33 ± 0.58 inds.m^-2^. *Serolis* sp. was the mobile organism that showed the highest mean density value (2.74 ± 0.63). However, it was due to a peak of abundance of 70 individuals recorded at 15 m. Other taxa, such as Porifera, Cnidaria and Ctenophora were rare with total abundances less than 10 individuals (Table 1).

**Fig 3:**
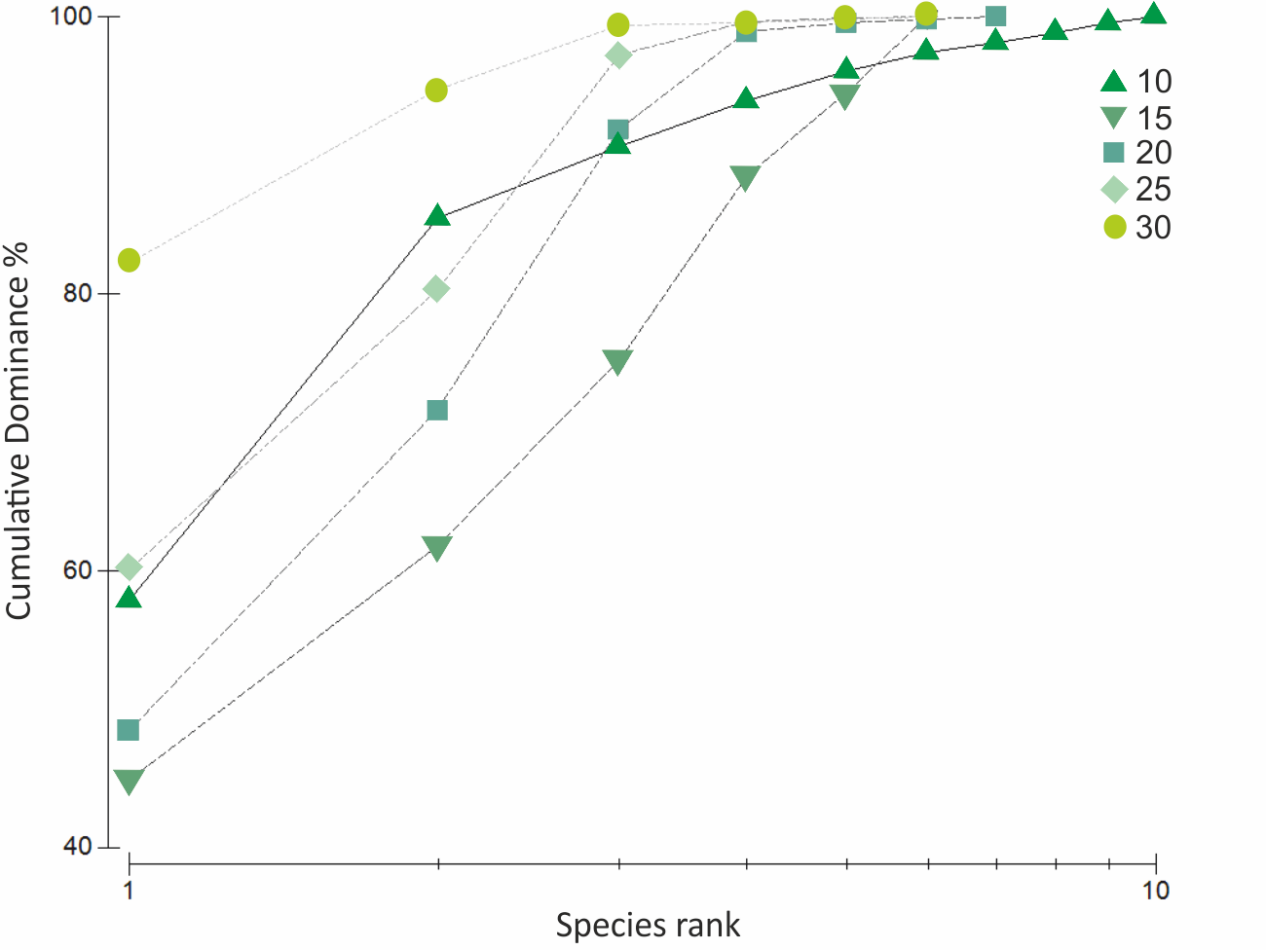
Cumulative k-dominance curves for each depths sampled in the new ice-free areas at Potter Cove. Each curve is based on abundance data (% cover).

Species composition was similar at all depths. Filter feeders comprised the largest trophic group, with the highest proportions at 25 m depth. Note that at 25 m the abundances of *C*. *verrucosa*, *M*. *pedunculata* and bryozoans were double or triple compared with the other depths (Table 2). The highest species richness (*S'*), 10 in total, was found at 10 m (Table 3). Diversity Shannon: *H´ (log base 10*) showed significant differences among depths (ANOVA, *F* = 23619.50, *p* <0.0001). However, these differences did not show a bathymetric pattern. This was also confirmed by k-dominance curves that showed a higher evenness at 15 m depth but also that curves crossed among them indicating that there is not a clear bathymetric pattern in diversity (Fig 3).

**Table 3:** Diversity indices across all depths sampled in the new ice-free areas at Potter Cove.

Depth (m)	*S'*	*J'*	*H'*
**10**	10	0.524	0.524
**15**	6	0.852	0.663
**20**	7	0.646	0.546
**25**	6	0.581	0.452
**30**	6	0.335	0.261

Diversity indices across all depths using species richness (*S'*), Pielou’s evenness *J'* and Shannon-Wiener index *H'* (log base 10).

**Fig 4:**
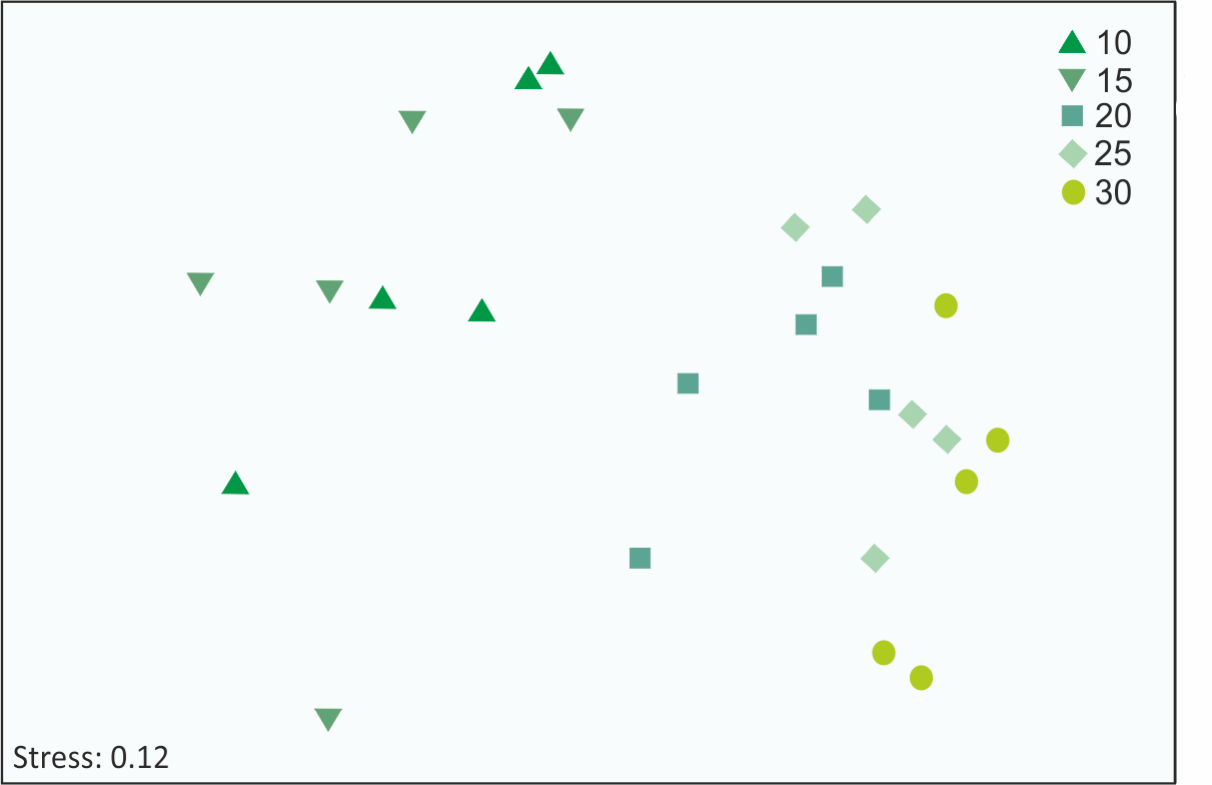
Non-metric multidimensional scaling biplot based on the Bray–Curtis similarity matrix for percentage cover of registered taxa (square-root transformed data).

Based on the Bray–Curtis similarity matrix for percentage cover data, dendrogram and MDS plots were constructed to represent the similarity among depths. Benthic assemblages were discriminated according to depth, and clustered in 2 main groups: one with shallower stations 10 and 15 m and the other with the deeper ones (20, 25 and 30 m). Groups were separated at high similarities indicating that assemblages from different depths were quite homogeneous and differed more in abundances than in composition. nMDS analysis showed samples in a single large group, but with a continuous bathymetric pattern along the main axis (Fig 4).

ANOSIM showed a slight but general significant difference between depths (Global ANOSIM *R* = 0.184, *p* < 0.001, 9999 permutations). Pairwise comparisons also showed significant differences (Table 4). SIMPER analysis confirmed larger dissimilarity between contiguous shallow depths (e.g. between 10 and 15 m), and smaller ones between deeper depths. The analysis also indicated that the main species responsible for the dissimilarities between different depths were bryozoans and the ascidians *C*. *verrucosa* and *M*. *pedunculata* (Table 5).

**Table 4:** ANOSIM R values of pairwise tests of adjacent depth stations in the new ice-free areas at Potter Cove.

	10	15	20	25
**15**	0.15			
**20**	0.0089	0.233		
**25**	0.263	0.411	0.088	
**30**	0.197	0.29	0.038	0.098

Global ANOSIM *R* = 0.184, *p* < 0.001, 9999 permutations.

**Table 5:** SIMPER analysis on square root transformed data showing the degree of dissimilarity between benthic assemblages.

	15	20	25	30
**10**	**46.52** *M*. *pedunculata* *C*. *verrucosa* Porifera *C*. *antarctica* *Serolis* sp. *O*. *validus*	**52.71** *Cellaria* sp. *M*. *pedunculata* *C*. *verrucosa* *O*. *validus* *C*. *antarctica*	**60.28** *Cellaria* sp. *C*. *verrucosa* *M*. *pedunculata* *O*. *validus*	**70.55** *Cellaria* sp. *C*. *verrucosa* *M*. *pedunculata* *O*. *validus*
**15**		**58.42** *Cellaria* sp. *M*. *pedunculata* *C*. *antarctica* *Serolis* sp. Porifera	**67.12** *Cellaria* sp. *C*. *verrucosa* *M*. *pedunculata* *Serolis* sp.	**72.49** *Cellaria* sp. *C*. *verrucosa* *C*. *antárctica* *M*. *pedunculata*
**20**			**32.89** *Cellaria* sp. *C*. *verrucosa* *M*. *pedunculata*	**37.45** *Cellaria* sp. *M*. *pedunculata* *C*. *verrucosa*
**25**				**34.02** *Cellaria* sp. *M*. *pedunculata* *C*. *verrucosa*

Taxa contributing most (75% cut-off) to the dissimilarity between benthic assemblages of each sampled depths.

## Discussion

Satellite time series and glaciological studies around Antarctica allow to calculate glacier area changes and to define also the ice front positions at specific times with a high precision [16]. In Potter Cove, the retreat of the Fourcade glacier was tracked since 1956 [36]. The knowledge of this glaciological data offers an excellent opportunity to determine precisely when a specific area was ice-free and therefore, available for benthic colonization. The long-term monitoring program carried out in Potter Cove provides one of the best platforms to study coastal ecosystem responses to environmental shifts and especially, colonization and early succession processes in this new available natural substrates. Indeed, most of the still scarce data on these processes caused by glacier retreat were produced in Potter Cove [21,22,46,47]. In this study, we report an assemblage dominated by mega-benthic sessile fauna, particularly ascidians and bryozoans with an almost absence of mobile species in the new ice-free soft bottom areas at Potter Cove. The prevalence of epibenthic filter feeders is common in this fjord and it seems to be also a common and extended feature of Antarctic benthic assemblages [19,48]. Nevertheless, the abundances found here were much lower than those reported in the close new rocky island, but still much higher than those reported in artificial settlement plates and natural disturbed substrates after similar exposure periods [22,27–29,34;49].

At all depths, taxonomic groups and species found it in the new ice-free areas are common taxa observed in similar substrata along the cove, which revealed high taxonomic similarity of epi-benthic composition in Potter Cove soft-bottom. Most of the major phyla and even classes were well represented; however, taxa found in the present study represented just ~35% of the full taxa list recorded on soft substrata in Potter Cove [19,50]. We found common species present along the cove, such as the starfish *Odontaster validus*, the isopod *Serolis* sp., the ctenophore *Lyrocteis flavopallidus*, bryozoans and solitary ascidians. The bivalve *Laternula elliptica*, the snail *Neobuccinum eatoni* and the sea anemone *Hormosoma scotti* were also present but they were very rare (e.g., only one specimen of snail was found at 10 m). Even when sponges were also present, they showed lower coverage than the one reported in the new island at Potter Cove [22]. The magnitude observed here was at least of one order less. Also, some other abundant species present in Potter Cove, such as the sea urchin *Sterechinus neumayeri*, the fast-growing sponge *Mycale (Oxymycale) acerata*, the nemertean worm *Parborlasia corrugatus*, the sea slug *Doris kerguelenensis*, the brittle star *Ophionotus victoriae* and the starfishes *Diplasterias brucei* and *Odontaster meridionalis* were not recorded on these new areas. Something to highlight was the absence of the pennatulid *Malacobelemnon daytoni*, in spite of its features (a species that inhabits soft substrates areas, tolerates high sedimentation and has high reproductive output and population turn-over [19,51–53]). The reproductive strategy of this species, rapid sexual maturation, more than one spawning per year and its dominance in heavily ice-impacted areas, together with its distribution expansion and increased population density in the glacial effluent zone of the inner part of Potter Cove, point to this pennatulid as an opportunistic species with a high colonization potential that nevertheless was not present in these new soft-bottom areas. We can not further speculate on the possible causes of this unexpected absence.

In general, ascidians are important members of the Antarctic benthic fauna. Particularly, in Potter Cove they are one of the dominant groups of the benthic assemblages, especially in soft bottoms areas [54]. In this study, we found just four out of 13 species reported in the soft bottom areas [54] where three of these species were solitary. Interestingly, a similar pattern was found in the rocky walls of the new island, where 5 out of 6 ascidian species reported were solitary [22]. The solitary ascidian species are in general broadcast spawners with external fertilization while colonial forms are brooders with internal fertilization. This favours a longer pelagic larval duration of solitary forms, allowing a major dispersal potential compared to colonial species [55]. Besides that, we also found ascidians at shallower than 10 m depth; it is the first area in Potter Cove where ascidians are present at such shallow depths in horizontal substrates.

Densities values of ascidians found here are among the higher reported in an Antarctic region including Potter Cove but still lower than those found in the new island or in outer of Marian Cove [19,22,56–59]. *Molgula pedunculata* is an opportunistic species that have been identified among macrofauna colonizing Antarctic ice scours or in settlement experimental panels [4,27,29,49,60]. It is an ubiquitous species that achieves high values of density and dominance. Here, *M*. *pedunculata* had a density of 34 inds.m^-2^ at 25 m, two and three times larger than those recorded in the inner part of Potter Cove [50] or in Larsen A South, 12 years after disintegration of Larsen A ice shelf [35]. Moreover, the common and widespread ascidians *C*. *verrucosa* and *C*. *antarctica* showed high abundances suggesting, as well, that these species can be considered as opportunistic and early colonizers species. Additionally, in Potter Cove more than 20 sessile macro-epibiotic taxa were found colonizing the tunics of the three above-mentioned ascidian species reported here [61]. Thus, ascidians could play an important role as secondary substrate and ecosystem engineers, providing suitable substrate for other species such as bryozoans, sponges and even also other ascidians. This increase benthic diversity on soft-bottoms areas and enriches habitat heterogeneity.

Diversity showed slight differences with depth but differed from the pattern exhibited in the “old” areas of the Potter Cove, and in the majority of Antarctic coastal areas, where a marked trend of diversity increment with increasing depth was reported [50,62–65]. Cluster and MDS analysis suggest two groups: one present at shallower depths between 10 and 15 m, dominated by ascidians and the presence of mobile fauna; and the other at deeper depths, dominated by ascidians and bryozoans. Multivariate analysis indicated high similarities between samples belonging to the same depth and also between contiguous depths. This was confirmed by ANOSIM and SIMPER analyses that showed high similarities with slight differences as indicated by R values but significant differences especially between shallow and deep zones.

### Structural driving factors for benthic assemblages

Two important factors shaping benthic assemblages are an increased frequency of iceberg scouring and the increment of sedimentation rates caused by the glacial run-off, which are highly affected by glacier retreat [31]. Due to the progressive glacier retreat, dramatic increases in both factors were observed at Potter Cove [19,38,41]. In general, the break-up of marine-terminating glaciers is accompanied by the formation of hundreds of floating ice pieces, especially during summer time. Icebergs beaching in shallow coastal areas cause disturbances of the coastal benthic fauna affecting bottom community structure and biodiversity [31]. However, the ice that falls from the glacier cliffs usually produce floating brash and growlers with a diameter no more than few meters [39, pers. obs.]. This size would not represent a disturbance factor for the substrate deeper than 10 meters. In the inner cove, divers frequently observed bottom marks, and shell fragments of bivalves that were apparently crushed by ice, these would be allochthonous icebergs entering to the fjord. However, in the newly ice-free areas sampled there were no traces of ice impact (pers. obs.). Due to the prevailing currents and winds in Potter Cove, external icebergs that enter to the fjord are usually found beached in the southern shore, while new ice-free areas are situated on the opposite coastline and so, probably sheltered of ice impacts deeper than 10 meters.

Climate-induced glacial meltwater leads to an increased occurrence of suspended particles in the sea, especially in areas close to the glacier front during the spring-summer months [38,40]. The assemblages structure or species performance in the glacio-marine Antarctic fjords have been recently evaluated, revealing heavy influence from this glacial meltwater processes [5,19,66–68]. In fact, the previous mentioned increased sediment runoff was recently highlighted as a potential causal factor to affect filter feeders physiology [51,67,69] and vertical distribution of macroalgae [47]; also is now recognized as a threat to coastal ecosystems [5,19,37]. Therefore, high concentrations of suspended particulate matter is an important driver for the species composition of glacially influenced fjords. This is consistent with the taxa that were more affected and favored by increased sedimentation rates in Potter Cove, these taxa exhibited different physiological responses to sediment load [19,41,51,67]. Despite the expected effects of high sedimentation rates, especially on benthic filter feeders, there is in Antarctic fjords an unique pattern of higher diversities in the inner areas of the fjords, where sedimentation is more intense than in the outer areas [5,19]. This is contrasting with diversity patterns observed in Arctic and temperate fjords [70–73]. Such pattern would indicate a higher tolerance to sedimentation of Antarctic epifaunal filter feeders than their Arctic and temperate counter parts. However, the sudden shifts recently reported in “old” assemblages in Potter Cove suggest that the system may cope with sediment load until a threshold is surpassed and the system collapses [19]. This could also explain the lower ascidian abundances in deeper areas, probably affected by a higher sediment load. In this context, the new assemblages, although relatively sheltered from ice action, are exposed to high sedimentation that could reach the threshold and produce the system collapse with the almost ascidians disappearance as occurred in other areas of the Potter Cove.

Two contrasting new ice-free areas were surveyed in Potter Cove. They showed similarities in terms of colonizing taxa but also marked differences in abundances and diversities. The vertical rocky walls of the new island exhibited higher abundances and diversities than the horizontal soft bottom areas reported in this work. Besides the different exposure time, which is almost negligible (ca. 6 years to ca. 4–5 years), substrate and slope could explain the differences between these new ice-free areas at Potter Cove. Indeed slope could be in this case more relevant than substrate, due most of the reported species are able to colonize both soft and hard substrates. In fact, slope can reduce ice disturbance and mainly sedimentation effects since lower amounts of inorganic matter per square meter would reach the vertical walls compared with horizontal substrates. Heavier inorganic particles fall faster than organic ones providing at the same depth, a higher proportion of organic fractions of seston in vertical substrate [74]. The relationship between organic and inorganic particles influences the feeding success of filter-feeding animals. Thus, this would favor the development of a higher secondary production in steep slopes than in horizontal areas subjected to high sedimentation as those found in the island and in the newly soft-bottoms at Potter Cove. In addition, the presence of a greater amount of sediments on the upper substrates may be negatively influencing the settlement of larvae [75].

The rapid warming along the WAP and extended glacier retreat in Antarctica are causing environmental shifts with the potential to severely affect benthic coastal ecosystems. On one hand driving to the collapse of benthic assemblages, but on the other one generating new areas for colonization. This in turn would drive to a positive feedback to the climatic change process by the organic carbon loss in the first case or to a negative feedback by the new carbon fixation in the second case. Our results are an important contribution to improve our current knowledge on these glacier-influenced ecosystems.
